# Transdiagnostic Cognitive Remediation Therapy for Patients with Eating Disorders: A Randomized Controlled Trial

**DOI:** 10.3390/nu17091460

**Published:** 2025-04-26

**Authors:** Tora Thorsrud, Odin Hjemdal, Linda Thorsen, Nadia Micali, Camilla Lindvall Dahlgren, Siri Weider

**Affiliations:** 1 Department of Psychology, The Norwegian University of Science and Technology (NTNU), 7491 Trondheim, Norway; odin.hjemdal@ntnu.no (O.H.); linda.thorsen@ntnu.no (L.T.); 2 Eating Disorder Unit, Department of Psychiatry, Levanger Hospital, Health Trust Nord-Trøndelag, 7600 Levanger, Norway; 3 Unit for Research and Innovation, Diakonhjemmet Hospital, 0370 Oslo, Norway; 4 Eating Disorders Research Unit, Ballerup Psychiatric Centre, Mental Health Services of the Capital Region of Denmark, 2100 Copenhagen, Denmark; nadia.micali@regionh.dk; 5 Department of Psychology, Oslo New University College, 0454 Oslo, Norway; camilla.lindvall.dahlgren@oslonh.no

**Keywords:** eating disorders, cognitive remediation therapy, transdiagnostic, neuropsychological function, RCT

## Abstract

**Background/Objectives**: Eating disorders (EDs) are associated with cognitive inefficiencies related to cognitive flexibility, central coherence, and inhibition. Transdiagnostic cognitive remediation therapy (TCRT) is a new adaption of cognitive remediation therapy aimed at addressing these difficulties across ED diagnoses. This study investigates the effects of TCRT as an adjunctive treatment for patients with EDs on cognitive and clinical outcomes. **Methods**: A randomized controlled trial compared the effect of 9 individual sessions of TCRT in conjunction with treatment as usual (TAU) compared to TAU only for patients with EDs and concurrent cognitive difficulties. Participants were assessed at baseline, post-treatment (12 weeks after baseline), and follow-up (6 months after post-treatment assessment). The outcome measures included neuropsychological tests and self-report questionnaires measuring cognitive difficulties and ED psychopathology. The analysis was in accordance with intention to treat principles. **Results**: Sixty patients with various ED diagnosis and concurrent cognitive difficulties were recruited. The TCRT group (*n* = 30) displayed significantly greater improvement in self-reported executive functioning, measured by the Behavior Rating Inventory of Executive Function—Adult version compared to the control group (*n* = 30). However, no superiority of TCRT was observed on performance-based measures of set shifting, central coherence, or inhibition. Moreover, there was no significant difference in improvement in self-reported ED psychopathology. **Conclusions**: TCRT may enhance compensatory mechanisms for cognitive inefficiencies rather than improve cognitive effectiveness or ED symptoms directly for patients with EDs and concurrent cognitive difficulties. Further investigation of how these impact everyday functioning may provide valuable insights into TCRT’s role in ED treatment.

## 1. Introduction

Eating disorders (EDs) are severe mental disorders characterized by significant disturbances in thoughts, behaviors, and emotions related to food, weight, and body image. The Diagnostic and Statistical Manual of Mental Disorders (DSM-5) [1] includes three primary diagnostic categories of EDs: anorexia nervosa (AN), bulimia nervosa (BN), and binge eating disorder (BED). Additionally, the residual category, other specified feeding and eating disorders (OSFED), includes patients with ED symptoms that fail to reach full threshold criteria of AN, BN, or BED. AN is characterized by persistent effort to lose weight, resulting in a significantly low body weight. In addition, individuals with AN experience an intense fear of gaining weight. Body image is disturbed, while body weight or shape exert undue influence on self-evaluation. Similarly, disturbed body image and weight/shape concerns are central features of BN. BN is further defined by recurrent episodes of binge eating, which is defined as consuming a substantially larger amount of food relative to what most individuals would eat under similar circumstances, accompanied by a sense of lack of control during the episode(s). Episodes of binge eating are followed by compensatory behaviors such as self-induced vomiting; misuse of laxatives, diuretics, and other medications; as well as fasting and/or excessive exercise. Patients with BED engage in frequent binge eating episodes but without engaging in compensatory behaviors. Although the diagnostic categories comprise specific behaviors and cognitions, there are several shared psychological and behavioral features across ED diagnoses [1]. Furthermore, it is not uncommon for individuals to migrate from one ED diagnostic category to another during their course of illness [2], underscoring the shared psychopathology.

EDs have profound negative impacts on the individual’s psychical health, psychosocial functioning, and emotional well-being [3]. Furthermore, EDs are associated with reduced quality of life and increased mortality rates [4]. Other psychiatric and somatic comorbidities such as anxiety disorders, mood disorders, attention deficit hyperactivity disorder, overweight, diabetes, and celiac disease often co-occur with EDs and may complicate treatment [5]. High treatment drop-outs rates and challenges in patient engagement, often due to ambivalence toward recovery, may further exacerbate the situation [6]. A recent meta-analysis estimated overall recovery rates across EDs as 46%, highlighting the urgent need for improved treatment outcomes for this patient population [2].

Neuropsychological deficits have been proposed as a possible transdiagnostic feature of various mental disorders [7]. Moreover, neuropsychological inefficiencies have been suggested as contributing and maintaining factors in EDs [8,9], with implications for treatment outcomes [10]. In recent years there has been an increase in studies on neuropsychological functions associated with ED psychopathology, including set shifting, central coherence, and inhibitory control [11]. Set shifting, often referred to as cognitive flexibility, is the capacity to alter behaviors or cognitions in response to contextual changes [12]. Weak central coherence refers to a processing bias that favors featural or detailed information at the expense of global integration of information [13]. Deficiencies in inhibitory control are associated with impulsivity [14]. Impulsivity is a multifaceted construct that can be described as engaging in rash actions in response to both positive and negative emotions, lack of planning before engaging in behaviors, sensation seeking, and limited capacity to maintain focus when distracted [15]. Even though patients with AN have been the primary focus of research on neuropsychological deficiencies in EDs, studies on individuals with BN or BED are increasing [11]. Evidence of weak central coherence is found in studies of both individuals with AN and BN [16,17,18,19], whereas difficulties related to set shifting are found in AN, BN, and BED [20,21]. Additionally, cognitive difficulties related to impulsivity are found in individuals with AN, BN, and BED [22,23].

Common psychopathology in patients with EDs, such as rigid thinking, strict rules regarding food and exercise regimes, rituals around weighing, and body checking, may be related to poor cognitive flexibility and over-attention to details (weak central coherence) [18]. Furthermore, impulsivity can also be linked to behaviors related to EDs such as disorganized eating patterns, binge/purge behaviors in response to negative emotions, or failure to maintain focus on weight gain or a meal plan over time [24].

Cognitive remediation therapy (CRT) is designed to improve cognitive functions and target different cognitive domains depending on the disorder being treated. There is evidence supporting its efficacy in both psychotic disorders [25] and mild cognitive impairment [26]. CRT for patients with EDs was initially developed as a conjunctive treatment for patients with AN, targeting cognitive inefficiencies related to set shifting and central coherence [27]. Emphasizing the thought process over content, CRT incorporates exercises, behavioral tasks, and reflective questioning aimed to increase awareness of thinking patterns and develop adaptive strategies. Tasks and exercises typically do not focus on food, weight, or body, allowing reflections on flexibility or bigger picture thinking without the added emotional activation these topics usually provoke in individuals with EDs.

Over the last decades, the outcome of CRT for EDs has been evaluated in case reports and case series, as well as in feasibility studies and randomized controlled trials (RCTs). Evidence supports the feasibility of CRT across different settings (individual and group) and age groups (children, adolescents, and adults) [28,29], whereas results from case series and feasibility studies have shown an association between CRT and cognitive improvement [29,30,31]. However, results from RCT studies have been inconsistent. A recent systematic review of reviews of CRT for AN reported contradictory findings regarding neuropsychological outcomes related to both set shifting and central coherence [32]. Moreover, a meta-analysis of RCTs of CRT revealed a small but not statistically significant effect on central coherence and no effect on improving ED symptoms or increasing BMI in patients with AN [33]. Improvements in set shifting after CRT, compared to control treatment, ranged from no differences to large differences (effect sizes *d* = 0.41–0.79) [34,35,36,37]. However, heterogeneity in neuropsychological measures complicates comparisons of findings related to set shifting [33]. The evidence for CRT for other EDs remains limited, with only two studies assessing the effect in patients with BN, OSFED, BED, or obesity and bingeing [35,38]. The results from these studies indicate an effect of CRT on ED psychopathology and ED-specific health-related quality of life in the sample that included patients with BN and OSFED and improved cognitive flexibility in the sample with patients with BED or obesity.

Qualitative studies indicate that both adult and adolescent patients, as well as parents and clinicians, rate CRT positively [39,40,41,42,43]. Several studies have reported that patients find CRT useful and that sessions promote awareness of thinking patterns and facilitate adapting new skills and strategies in daily life [40,41,44]. Moreover, CRT has been associated with lower drop-out rates compared to control conditions [33,45] and has been suggested as a means to engage patients in treatment [28]. Research has also shown that patients with poor baseline set-shifting ability benefit more from CRT in terms of improved quality of life than patients without these difficulties [35], indicating a need for further investigation of the effect of CRT in patients with EDs who display cognitive difficulties at baseline.

Given the overlap in psychopathology and cognitive difficulties across ED diagnoses, it has been suggested that CRT could potentially be beneficial for patients across the entire spectrum of EDs [17,18]. Transdiagnostic CRT (TCRT) is a novel adaption of CRT developed to address cognitive inefficiencies across ED diagnoses. While retaining the principles and structure from previous manuals of CRT for AN [46,47,48] and CRT for obesity [38], modifications were made to adapt the manual for a transdiagnostic ED sample [49]. In addition to targeting difficulties related to set shifting and central coherence, modules addressing impulsivity and planning have been incorporated. Furthermore, the TCRT manual offers several interchangeable modules related to the different domains, allowing for treatment customization based on patient and therapist evaluations. This approach aims to more effectively address each patient’s unique cognitive challenges. In a recent qualitative study from our group, TCRT was rated positively, as patients described that it raised awareness of thinking patterns and offered a starting point for making changes and using new strategies [50]. However, larger treatment studies (RCTs) are needed to determine the effect of TCRT. The current study is the first to quantitively investigate the effect of TCRT on neuropsychological functioning and ED psychopathology.

The aim of the current study was to explore the effect of TCRT on ED psychopathology and neuropsychological functioning when delivered as an adjunctive treatment to treatment as usual (TAU) in a transdiagnostic sample of ED patients with concurrent cognitive difficulties.

## 2. Materials and Methods

### 2.1. Study Design and Procedure

**Figure 1:**
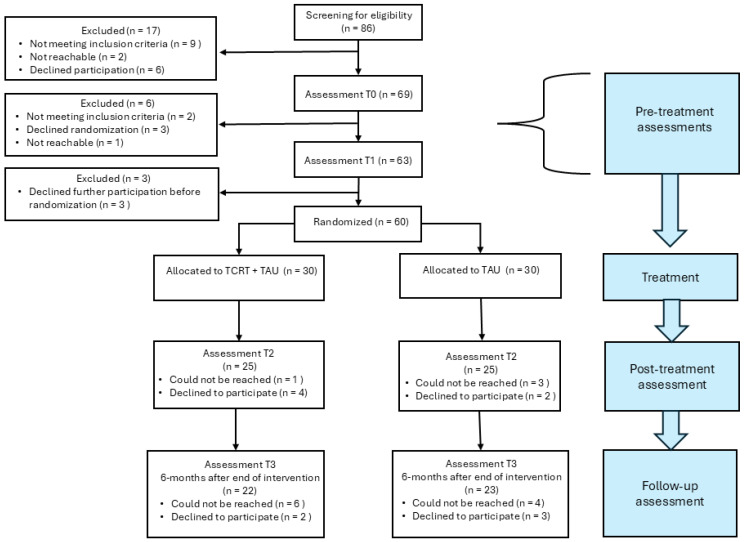
Study phases and patient flow.

The study was a randomized parallel study designed to investigate the effect of TCRT as a conjunct to TAU for patients with any type of ED and concurrent cognitive difficulties. The study consisted of four phases: pre-treatment assessment (T0 + T1), treatment, post-treatment assessment (T2), and follow-up assessment (T3). During the first pre-treatment assessment (T0), patients were screened for cognitive difficulties using standardized neuropsychological tests and questionnaires. Patients who displayed cognitive difficulties were invited to participate in the second pre-treatment assessment (T1) and in the RCT. The T1 assessment was conducted within a 2-week timeframe of T0. Following assessment at T1, patients were randomly allocated to receive either TAU + TCRT or TAU only. Assessments with neuropsychological tests and self-report questionnaires took place post- treatment (T2: 12 weeks after T0) and at follow-up (T3: 6 months after T2). Details of the measures used at the different assessment times are found in Table 1. A study flow chart is presented in Figure 1. Assessors were clinical (neuro)psychologists or advanced psychology students who had undergone extensive training in neuropsychological testing supervised by a clinical neuropsychologist. All adverse events were registered.

### 2.2. Participants and Recruitment

Three specialized ED units in Norway participated in the study: the Regional Eating Disorder Unit at Levanger Hospital; the Eating Disorder Unit at St. Olavs Hospital, Trondheim; and the Regional Eating Disorder Unit at Oslo University Hospital, Oslo. Patients at these units received general information about the study through information pamphlets, posters, or members of the treatment team. If a patient expressed interest in the study, a member of the research team contacted the patient to provide more detailed information about the study. This approach ensured that patients did not feel pressured by the treatment team to participate. Initial eligibility to the study was also assessed at this time. For a comprehensive overview of the inclusion and exclusion criteria, see Table 2. After receiving detailed information about the study, patients were asked to provide written consent if they wished to participate. Eligibility for randomization into the treatment study was determined based on the T0 assessment, as described under procedures. In Figure 1, we present the study flow and patient allocation.

### 2.3. Randomization and Blinding

A computerized program developed by the Unit for Applied Clinical Research, Faculty of Medicine, Norwegian University of Science and Technology, Trondheim, was used for randomization. Randomization was stratified by ED diagnosis to ensure equal representation of diagnoses in the two groups. For this purpose, three groups were defined; 1: AN + OSFED-AN, 2: BN + OSFED-BN, or 3: BED + OSFED-BED. Furthermore, given that the majority of patients who receive inpatient treatment in specialized ED units are patients with AN, this stratification helped to ensure a more balanced distribution of TAU settings across the two groups. Assessors were blind to participant treatment allocation.

### 2.4. Intervention

#### 2.4.1. Transdiagnostic CRT

Patients received 9 weekly sessions of individual TCRT, lasting approximately 45 min. The transdiagnostic CRT manual [49] was based on the previous CRT manuals developed by Tchanturia [27,46,51] and Dahlgren [47] and were adapted to a transdiagnostic sample in collaboration with professor Camilla Lindvall Dahlgren. The manual offers 9 structured sessions incorporating cognitive and behavioral exercises designed to enhance metacognitive awareness and challenge cognitive inefficiencies in the following domains: (1) planning–impulsivity, (2) flexibility–rigidity, and (3) central coherence–attention to detail. Each exercise was followed by questions and dialog designed to promote reflection on thinking process and exploration of alternative strategies. Efforts were made to connect in-session exercises to everyday life through these reflective discussions. The first four sessions followed a set structure, each focusing on one of the three domains in addition to socialize the patient to the treatment. In the fifth session, an interim evaluation was conducted to assess treatment progress and patient preferences regarding specific exercises and therapeutic domains. Based on this evaluation, tasks for the remaining four sessions were selected from various modules in the treatment manual to tailor the treatment to the patient’s specific needs. A more detailed account on the treatment implementation is provided in Thorsrud, 2024 [50].

#### 2.4.2. Therapist Training and Treatment Fidelity

Treatment was delivered by a clinical psychologist, advanced psychology students, or trained research nurses. All TCRT therapists had attended workshops in CRT conducted by either Professor Kate Tchanturia or Professor Camilla Lindvall Dahlgren. For each patient, therapists were exclusively involved in either TCRT or TAU, ensuring no overlap in treatment provision. Sessions were videotaped to assess treatment fidelity by the 5th author (C.L.D.). Randomly selected videos from all TCRT therapists were assessed and written feedback was provided.

#### 2.4.3. TAU

All units were part of the public health care system in Norway and provided specialized treatment for EDs within their respective regions. The Norwegian Directorate of Health has established national guidelines for treatment for EDs [52], which mandate that patient care should focus directly on the ED symptomatology. This includes normalization of weight in the case of underweight, reduction of binging and purging, and normalization of other ED-related thoughts and behaviors. For patients with BN, cognitive behavioral therapy is specifically recommended. Although these guidelines are not legally binding, any deviations must be documented to ensure the maintenance of quality, good practice, and equality in service provision. The type of TAU, whether outpatient, inpatient, or day-treatment, was recorded for each patient.

### 2.5. Measures

#### 2.5.1. Demographics and Diagnostic Variables

Interviews covering topics including age, educational level, duration of ED, duration of ED treatment, height, weight, nadir weight (lowest ever weight on current height), and use of psychotropic medication (type) were conducted by a clinical psychologist or advanced psychology students. All ED diagnosis were assessed by clinical psychologists using the Norwegian version of the Eating Disorder Assessment for DSM-5 (EDA-5) [53].

#### 2.5.2. Self-Report Questionnaires

Symptoms of depression and anxiety: The Beck Depression Inventory (BDI) [54] and Beck Anxiety Inventory (BAI) [55] were used to assess symptoms related to depression and anxiety in the sample. The BDI is a widely used 21-item self-report questionnaire in which patients rate the occurrence and severity of depressive symptoms within the last two weeks. Higher scores indicate higher symptom severity. The BAI was used to assess symptoms related to anxiety. Patients rate the occurrence and severity of various cognitive and physiological symptoms of anxiety in the last two weeks on a scale from 0 to 3. A higher score indicates higher severity of symptoms.

Eating disorder symptomology: Psychopathology and symptoms specific to EDs were assessed using the Eating Disorder Inventory-3 (EDI-3) [56], the Eating Disorder Examination Questionnaire (EDE-Q) [57], and the Eating Disorder Flexibility Index (EDFLIX) questionnaire [58]. The EDI-3 is a 91-item self-report inventory that offers 12 subscales assessing both ED-specific as well as broader psychological traits and symptoms associated with EDs. The subscales Drive for Thinness, Bulimia and Body Dissatisfaction cover core ED symptomology such as preoccupation with weight and body shape, tendencies toward binge eating, and body dissatisfactions. A higher score indicates higher levels of symptoms. The EDE-Q is a widely used 31-item self-report questionnaire that is used for the assessment of severity of ED symptoms. Patients are asked to rate attitudinal features as well as ED-related behavior in the last 28 days. A global score is calculated based on the average of four indexes (Dietary Restraint, Eating Concern, Weight Concern, and Shape Concern). A higher score indicates higher levels of symptoms. A clinical cut-off score of 2.5 on the global EDE-Q score has been suggested in a Norwegian sample [59]. The EDFLIX is a measure of cognitive and behavioral flexibility with 36-items assessing general mental flexibility and flexibility related to food, exercise, weight, and shape. An EDFLIX total score is derived by summing all items. A higher score indicates higher levels of flexibility. A clinical cut-off score of 136 has been suggested in a Norwegian adult sample [58].

#### 2.5.3. Self-Reported Neuropsychological Assessment

Executive functions: The Behavior Rating Inventory of Executive Function—Adult version (BRIEF-A) [60] is designed to assess executive functioning in everyday life. It offers nine subscales, which are summarized into two main indexes: the Behavioral Regulation Index (BRI; includes the subscales Inhibit, Shift, Emotion Control, and Self-Monitor) and the Metacognition Index (MI; includes the subscales Initiate, Working Memory, Plan/Organize, Task Monitor and Organization of Material). In addition, it offers a comprehensive measure of overall executive functioning, termed the Global Executive Composite (GEC).

#### 2.5.4. Performance-Based Neuropsychological Assessments

Intellectual ability: All patients completed four subtests (block design, matrix reasoning, similarities, and vocabulary) of the Wechsler Adult Intelligence Scale 4th edition (WAIS-IV) [61] enabling the calculation of the General Ability Index (GAI) as an estimate of intellectual functioning. The GAI was chosen as its calculation requires fewer subtests than the full-scale IQ index from the WAIS-IV, thus reducing patient burden. Additionally, it provides an indication of premorbid functioning as it relies less on working memory and processing speed.

Central Coherence: The Rey Complex Figure Test (RCFT) [62] was used to assess central coherence. In this task, the patient is required to replicate a complex figure stimulus using pencil and paper. The scoring system developed by Lopez, 2009 [63] was used to evaluate both the order in which the elements are drawn (global or detail oriented) as well as the style (coherent or fragmented) through the Order of Construction Index and Style Index. Together, these two indices are used to calculate the Central Coherence Index (CCI). A clinical neuropsychologist (S.W.) was always consulted when scoring the RCFT as a measure of quality control. A higher CCI score indicates better central coherence with a more global processing ability.

Set-shifting tasks: Set shifting was measured with the computerized 128-card version of the Wisconsin Card Sorting Test (WCST) [64]. In this task, the patient is presented with a series of cards depicting symbols in various colors, shapes, or numbers. The patient is required to match the cards according to unknown classification rules and is given feedback after each trial. After 10 correctly matched cards, the classification rule changes unbeknownst to the patient, requiring them to adapt to the new rule based on the feedback they receive. Perseverative responses and perseverative errors are commonly used as indicators of set-shifting difficulties, with higher scores reflecting greater difficulties with set shifting.

Inhibition: Condition 3 on the Color Word Inference Task (CWIT) from the Delis–Kaplan Executive Function System (D-KEFS) [65] measures inhibition difficulties. In this task, the patients are presented with color words printed in incongruent colored ink. The patient is asked to name the color of the ink and thus suppress an automatic response (reading the printed words). The time required to complete the task is recorded, with longer completion time reflecting greater inhibition difficulties.

#### 2.5.5. Outcomes

Primary outcome measures were defined as executive functioning measured by the BRIEF-A, set-shifting ability measured by WCST, central coherence measured by RCFT, inhibition measured by condition 3 in CWIT, and ED symptomatology measured by the EDI-3 and the EDE-Q. Secondary outcomes were defined as performance on additional neuropsychological tests related to set shifting, decision making, planning, and inhibition, as well as self-report questionnaires measuring symptoms of depression, anxiety, and quality of life. These secondary outcomes were analyzed separately.

### 2.6. Statistical Analysis

The baseline data were analyzed using SPSS version 30.0.0. Independent sample *t*-tests, Mann–Whitney U tests, or Chi-square tests were used to examine group differences at baseline as appropriate. One-sample *t*-tests or Wilcoxon signed-rank tests were used to compare performance on neuropsychological self-reported or performance-based measures to normative scores for the sample at baseline. For this purpose, raw scores were transformed to standardized scores (*t*-scores or scaled scores). Age-based normative means were gathered from the test manuals for the BRIEF-A, WCST, and CWIT. Higher *t*-scores on the performance-based measures indicated better performance, whereas higher *t*-scores on self-reported cognitive difficulties indicated higher levels of difficulties. No age-based normative data were available for the CCI and EDFLIX. As an alternative, mean scores based on healthy controls published by Lang, 2016 [66] were used for the CCI. Since the scores on the EDFLIX were not normally distributed and the necessary normative values (medians) to perform non-parametric tests have not been published, no comparison was performed for this measure. Effect sizes were calculated using Cohen’s *d*, when using parametric tests on baseline comparisons, with *d* = 0.2 indicating a small effect, 0.5 indicating a medium effect, and 0.8 indicating a large effect [67]. All other analyses were performed in STATA IC version 18.0. To reduce bias in the results, the analyses were conducted in accordance with the intention-to-treat (ITT) principle (including all randomized participant regardless of the amount of treatment they received). Differences in symptoms over time were assessed using planned contrast within multilevel modeling, which allows for estimation of changes in repeated measures over time despite missing data. Missing data were handled using full information maximum likelihood estimation. All models included fixed effects of time and group. In the coefficients model for outcomes, we investigated the extent to which, after having received treatment, the gains in both groups were sustained or improved at the 6-month follow-up. We computed a time effect, representing the difference in the change over time for the groups. The linear combination of coefficients function was used to create model-predicted means and all planned contrasts. The model-predicted means and SE values are presented for continuous variables at all timepoints from pre-treatment to follow-up.

### 2.7. Ethical Approval and Considerations

The study was approved by the Regional Committee for Medical and Health Ethics of Central Norway (reference 2018/1182) and the Data Access Committee (DAC) at Levanger Hospital (reference 2018/1740). The trial was pre-registered at clinicaltrials.gov (ID: NCT03808467).

All participants were informed that participation would not affect their current treatment and that they could withdraw consent at any time. All participants gave written informed consent. According to Norwegian legislation, individuals over the age of 16 can consent to participation research trials without parental involvement. However, extra care was taken when presenting information about the study and obtaining consent from patients under the age of 18. Patients in the control group were given the option to receive 9 sessions of TCRT after the 6-month follow-up.

## 3. Results

### 3.1. Baseline Characteristics of the Sample and Treatment Delivery

Potential participants (*n* = 86) were recruited between March 2019 and August 2023. In total, 60 patients were randomized, excluding 26 based on the inclusion and exclusion criteria. The T1 assessment was conducted on average 7.2 days after the T0 assessment. Figure 1 shows the patient flow throughout the study, and Table 3 and Table 4 summarize the participant characteristics at baseline. No significant differences in clinical or demographic variable were observed between the TCRT group and the control group at baseline. Additionally, there were no significant differences between the two groups in terms of ED diagnosis, type of TAU treatment (inpatient or outpatient), or use of psychopharmacological medication (yes/no).

At baseline, the sample exhibited better performance on the WCST with significantly fewer perseverative errors and perseverative responses compared to the normative scores. Additionally, significantly weaker central coherence measured by the CCI was observed. No significant difference from the normative scores was observed on the CWIT (condition 3). The sample also reported significantly higher levels of cognitive difficulties on the BRIEF-A. Comprehensive details on the cognitive function of the sample compared to the normative scores are presented in Table 5.

Seven patients in the TCRT group received ≤ 3 sessions of TCRT due to the severity of other symptoms (*n* = 4), restrictions related to COVID-19 (*n* = 2), or lack of interest in continuing sessions (*n* = 1). The remaining 23 patients in the TCRT group received on average 8.9 sessions. At the time of the T2 assessment, 48 patients were still in treatment at their respective ED units and 2 patients were no longer in treatment, with no significant difference between the two groups (*X*(1) = 0.001, *p* = 0.976). Information about TAU at T2 was missing for 10 patients as they did not participate in the T2 assessment. Due to restrictions related to the COVID-19 pandemic, re-testing was delayed for several patients. A Mann–Whitney U test was conducted to compare the delay in T2 testing between the two groups. The results indicated a significant longer delay in the TCRT group (*M* = 26.7 days) compared to the control group (*M* = 11.8 days, *U* = 169.5, *Z* = −2.78, *p* = 0.005). However, there was no significant difference in delay of time of assessment at T3 (*U* = 276.0, *Z* = −0.732, *p* = 0.464).

### 3.2. Treatment Outcomes

The primary outcomes using the planned contrast in the multilevel model analysis revealed non-significant changes from the pre-treatment, post-treatment, and 6-month follow-up assessments on performance-based neuropsychological measures (WCST, RCFT, and CWIT) as well as assessments related to ED psychopathology (Table 6). Furthermore, the analyses showed a significant change from pre-treatment, post-treatment, and 6-month follow-up assessments on self-reported cognitive difficulties measured by the BRIEF-A subscales BRI and GEC.

### 3.3. Adverse Events

During the trial, three cases of adverse events were reported: one case of an expedited scheduled hospitalization and two cases of increased suicidal ideation. After thorough evaluation, all cases were determined to be clearly unrelated to study participation.

## 4. Discussion

The aim of the current study was to investigate the effect of TCRT on cognitive function and ED psychopathology in a transdiagnostic sample of ED patients with concurrent cognitive difficulties. This is the first study to explore the effects of this novel adaption of CRT for EDs. The study revealed three main findings.

Firstly, no significant difference in improvement in performance-based measures of set shifting, central coherence, or inhibition were observed when comparing the TCRT group with the control group. These findings are consistent with several previous RCTs that also did not find CRT to improve performance-based measures of set shifting and central coherence compared with control conditions [35,36,68]. However, there are some RCTs that have found an effect of CRT on performance-based measures; one study reported a short term effect on set shifting assessed with the Trail Making Test, but not with the WCST [69], while another found a significant effect of CRT in young people with AN [70]. Importantly, our sample exhibited significantly fewer perseverative responses and perseverative errors compared to the normative scores at baseline. Consequently, the potential for improvement in performance on this measure may be limited as the group already displayed good set-shifting ability on the task.

A second finding is that there was no difference in improvement in self-reported measures of ED psychopathology between the TCRT group and the control group. This results aligns with the meta-analysis performed by Hagan, 2020 [33], which similarly identified no significant impact of CRT on ED symptoms at end of treatment.

Thirdly, the TCRT group demonstrated significant improvement in self-reported executive function difficulties compared to the control group. This difference was observed in the BRIEF-A subscale BRI, as well as the overall composite score (GEC). Improvement in self-reported executive functioning, as measured by various versions of BRIEF, have been documented in within-subject studies involving adolescent patients with AN [71,72]. To the best of our knowledge, the current study is the first RCT to investigate the effects of CRT on self-reported executive function, as measured by the BRIEF-A.

The impact of TCRT on self-reported cognitive difficulties, as opposed to performance-based measures, warrants some further consideration. Although several studies failed to demonstrate a superior effect of CRT on performance-based measures, this contrast with patients’ feedback in qualitative studies. Qualitative evaluations of CRT suggest that patients find the treatment useful for increasing awareness of their thinking patterns as well as adopting new problem-solving techniques [32]. In the qualitative study related to the current RCT, patients described both improved awareness of thinking style and behavioral changes attributed to TCRT [50]. Interestingly, another RCT also found a discrepancy between performance-based neuropsychological measures and self-reported changes in thinking patterns through thought record analysis for adults with EDs [36]. Considering these findings, there is some indication that TCRT may have effects related to cognitive difficulties that are not captured by performance-based neuropsychological measures.

Discrepancies between performance-based and self-reported measures of cognitive functions have been identified in EDs [73], other clinical populations, and non-clinical groups [74,75]. The BRIEF-A has been suggested to offer a more ecologically valid measure of daily-life executive functioning compared to performance-based measures [73,76]. However, the BRIEF-A rating may also be related to emotional distress [77] and it has been suggested to capture emotional aspects of executive functioning [78]. It has been posited that performance-based and self-reported measures of cognitive functions assess different underlying constructs, capturing distinct levels of cognitions [74]. Specifically, performance-based measures have been proposed to reflect the efficiency of cognitive abilities, whereas self-reported measures are more related to success in goal pursuit. Success in goal pursuit requires cognitive effectiveness but is likely affected by additional factors, such as emotional distress. This may, in part, explain some of the association between BRIEF-A scores and emotional distress.

Cognitive training programs are often considered to have restorative or compensatory mechanisms, or both. In this context, restorative mechanisms refer to interventions that improve the efficacy of cognitive functions whereas compensatory mechanisms focus on increasing awareness and compensatory strategies [79]. Restoration or enhancement of specific cognitive functions or performance on specific tasks would require task-specific training [80]. However, the benefits of training-specific tasks are often limited to those tasks (and sometimes similar tasks) and do not readily generalize across settings or tasks [80]. Thus, efficient training in sorting cards with changing rules may improve performance on the WCST but it does not necessarily enhance set-shifting ability in other contexts. The focus in TCRT is on metacognitive awareness while exploring and adapting alternative strategies. One objective is for patients to apply insights and strategies from sessions to everyday situations, thereby generalizing these skills from the therapeutic setting to daily life. In light of this, limited impact of TCRT on performance on neuropsychological tests may be expected. The improvement in self-reported executive function, contrasted by the lack of improvement in performance-based measures, suggests that TCRT may primarily have a compensatory rather than a restorative effect. However, while TCRT may enhance everyday cognitive functioning or goal achievement, it does not appear to impact ED psychopathology directly according to the findings of the current study.

Examining the effect of TCRT as a concurrent treatment within a naturalistic TAU setting, incorporating multiple treatment sites, adds value to the study by making the results more applicable to real-world clinical settings. However, this approach also presents challenges and important considerations for interpreting the results. Since patients receive their TAU from various ED-units, there may be considerable differences in treatment content or intensity, despite the existence of national guidelines for ED treatment. Efforts were made to record the intensity of TAU by providing patients with logs to register TAU treatment or calling patients once a week to gather information about TAU frequency. However, this proved challenging as the majority of patients did not adhere to registration protocol or respond to phone inquiries. Although the type of TAU (inpatient or outpatient) was recorded, a more detailed documentation of TAU would have strengthened the study. We anticipate the most variability of treatment intensity in the outpatient settings, as inpatient treatment in the included units adhered to a structured program including individual treatment, group treatment, and meal support. There was no significant difference in patient distribution in inpatient or outpatient TAU treatment across the two groups. Treatment fidelity was assessed by the 5th author (C.L.D.), rather than an independent expert on CRT, which is a limitation of the current study.

A notable strength of the current study is the utilization of intention-to-treat analysis to evaluate the effect of TCRT, as it minimizes bias in the findings. The relatively short follow-up (6 months post-treatment) might limit insights into long-term treatment effects. A broad range of outcome measures were used to assess both cognitive and psychopathological outcomes. However, the same neuropsychological tests were used at all timepoints. Using alternative versions could potentially limit the practice effects. Given that both groups were assessed an equal number of times, similar practice effects are expected, thereby minimizing their impact on differences in outcomes.

Additionally, the rigorous clinician validation of ED diagnoses enhances the quality of the study. Including patients with OSFED in clinical trials is crucial, as this group is often excluded from treatment studies despite constituting a large portion of patients that are severely affected by their EDs. The majority of included patients were diagnosed with AN or OSFED-AN, limiting the transdiagnostic scope of the study. Better representation of patients with BN or BED would have been preferable. The skewness in diagnoses was unintentional and likely due to patient recruitment from specialized ED units to ensure comparable TAU treatment. Unfortunately, patients with BN and BED are less likely to receive specialized ED treatment in Norway compared to patients with AN. Consequently, the number of potential participants with a BN or BED diagnosis were lower compared to those with AN. In addition, the sample of the current study was relatively small. Consequently, subtle yet clinically relevant effects of TCRT might have been missed due to limited statistical power.

There was a significant difference in time between assessments, with the TCRT group having longer intervals between pre-treatment and post-treatment assessments. Longer intervals could mean that the TCRT group was less affected by test–retest or training effects on the performance-based neuropsychological tests, potentially representing a disadvantage for performance compared to the control group. Time is an important factor in recovery. Longer time intervals between assessments could lead to a difference in changes in psychopathology not related to the TCRT treatment but rather to longer TAU treatment or other factors. Given the association between BRIEF-A and emotional distress, it could be argued that significant changes in BRIEF-A scores may reflect changes in ED psychopathology rather than improved cognitive functions. However, as previously mentioned, there was no difference in ED psychopathology between the two groups observed over time. Regardless, we cannot rule out that differences in delays in post-treatment assessments between the TCRT and control groups might have had a confounding impact on the outcomes.

Future research investigating the effect of TCRT should include power analysis to ensure sufficient statistical power. Furthermore, better representation of patients with BN, BED, or atypical EDs should be prioritized. This will, in addition to a larger sample, enable subgroup analysis based on symptomology and other clinical characteristics that can refine treatment targeting and inform future adaptions of the manual. Additionally, potential long-term outcomes related to ED psychopathology and quality of life should be investigated to better understand the potential benefits of TCRT. Exploring adaption of TCRT for tele-health delivery could improve outreach and accessibility.

## 5. Conclusions

The TCRT group displayed significantly greater improvement in self-reported executive function difficulties but not performance-based measures of cognitive functions or self-reported ED psychopathology compared to the control group. The findings suggest that TCRT may strengthen compensatory mechanisms for cognitive inefficiencies rather than directly improving cognitive effectiveness or ED psychopathology. For clinicians, these results imply that TCRT can be a useful tool for patients with EDs and cognitive inefficiencies in developing compensatory strategies that will help them to improve everyday functioning. Future investigation of how this impacts everyday functioning, long-term ED outcomes, and quality of life will provide valuable insights into the role of TCRT for patients with EDs.

## Figures and Tables

**Table 1:** Measures and assessment times.

	Baseline Assessment		Post-Treatment Assessment	Follow-Up Assessment
	T0	T1	T2	T3
Self-report assessments				
EDE-Q	X		X	X
BDI	X		X	X
BAI	X		X	X
BRIEF-A	X		X	X
Neuropsychological test assessments				
WAIS-IV	X			
WCST	X		X	X
RCFT	X		X	X
CWIT	X		X	X
TMT		X	X	X
IGT		X	X	X
Tower Test		X	X	X
CPT-3		X	X	X

EDE-Q = Eating Disorder Examination Questionnaire, BDI = Beck Depression Inventory, BAI = Beck Anxiety Inventory, BRIEF-A = Behavior Rating Inventory Of Executive Function—Adult version, WAIS-IV = Wechsler Adult Intelligence Test Wechsler Adult Intelligence Test, 4th edition, WCST = Wisconsin Card Sorting Test, RCFT = Rey Complex Figure Test, CWIT = Color Word Interference Test, TMT = Trail Making Test, IGT = Iowa Gambling Test, CPT-3 = Conner’s Continuous Performance Test-3.

**Table 2:** Inclusion and exclusion criteria.

Inclusion Criteria to Baseline Assessment (T0)	Exclusion Criteria to Baseline Assessment (T0)	Inclusion Criteria to T1 and Randomization
Female sex Age 16–36 Understand and speak Norwegian Currently in ED treatment Providing written consent	History of congenital or acquired brain injury (except concussions) Active substance abuse Psychosis IQ < 70	Displaying cognitive difficulties defined as performance 1 SD below the normative average on either: (a) Total Errors, Perseverative Responses, Perseverative Errors, and/or Learning to Learn from the WCST and/or on the RCFT copy condition or Q-score and/or on condition 3 and 4 on the CWIT from D-KEFS or (b) Inhibit, Shift, Plan/Organize, and Global Executive Composite indexes of the self-reported measures from the BRIEF-A Providing consent to randomization

BRIEF-A = Behavior Rating Inventory of Executive Function—Adult version, CWIT = Color Word Inference Test, D-KEFS = Delis–Kaplan Executive Function System, ED = Eating disorder, WCST = Wisconsin Card Sorting Test.

**Table 3:** Comparison of clinical and demographic variables between the TCRT and control group at baseline.

	TCRT Group				Control Group							
Variable	*n*	Mean	Median	SD	*n*	Mean	Median	SD	*z* ^a^	*t* ^b^	*p*	*d*
Age (years)	30	23.83	23	4.85	30	24.33	24.00	3.93	−0.82		0.41	
BMI	29	18.66	17.70	2.88	29	18.21	17.30	3.10	−0.84		0.39	
DOI (years)	30	7.77	7	6.08	30	7.55	7.50	3.91	−0.45		0.65	
DOT (years)	30	4.42	4	3.78	30	5	4.00	4.35	−0.49		0.62	
Education (years)	30	13.77	13	2.05	30	13.73	13.00	1.74	−0.29		0.77	
GAI	30	100.3	98.5	15.23	30	106.57	106.50	11.48		−1.80	0.08	−0.47
EDE-Q	29	4.17	4.57	1.19	30	3.92	4.18	1.15	−0.88		0.38	
BDI	29	34.31	37	10.90	28	30	30.00	11.70		1.45	0.15	0.38
BAI	28	26.71	24	12.02	28	22.43	21.50	9.67		1.47	0.15	0.39

BAI = Beck Anxiety Inventory, BDI = Beck Depression Inventory, BMI = Body Mass Index. DOI = Duration of Illness, DOT = Duration of Treatment, EDE-Q = Eating Disorder Examination Questionnaire, GAI = General Ability Index, ^a^ result Mann–Whitney U test ^b^ result of independent sample *t*-test.

**Table 4:** Comparison of categorical clinical and demographic variables at baseline.

	TCRT Group (*n* = 30)	Control Group (*n* = 30)	Statistics
Eating disorder subtype			
AN	17	21	*X*(4) = 3.877, *p* = 0.423
OSFED AN	9	4	
BN	2	3	
OSFED BN	1	2	
OSFED BED	1	0	
Medication			
Yes	25	22	*X*(1) = 0.88, *p* = 0.347
No	5	8	
TAU			
Inpatient	16	14	*X*(1) = 0.267, *p* = 0.606
outpatient	14	16	
Socioeconomic status			
School/study	9	11	*X*(2) = 0.38, *p* = 0.826
Employed	3	2	
Sick leave/disability	17	17	

AN = Anorexia nervosa, BN = Bulimia nervosa, BED = Binge eating disorder, OSFED = Otherwise specified eating disorders, TAU = Treatment as usual.

**Table 5:** Cognitive function at baseline for the entire sample (*n* = 60) compared to normative scores.

	Study Sample		Standardized Norms					
	**Mean**	**SD**	**Mean**	**SD**	***z* ^b^**	***t* ^a^**	***p***	***d***
BRIEF-A ^a^								
BRI	63.38	10.80	50	10		9.60	<0.001 **	1.24
MI	63.73	10.65	50	10		9.99	<0.001 **	1.29
GEC	64.60	10.79	50	10		10.48	<0.001 **	1.35
WCST ^b^								
Perseverative responses	55.27	13.98	50	10		2.92	0.005 *	0.38
Perseverative errors	53.92	12.65	50	10		2.40	0.020 *	0.31
CWIT ^b^								
Condition 3	9.80	3.11	10	3	0.13		0.898	
RCFT ^b^								
CCI	1.21	0.37	1.40	0.36		−3.98	<0.001 **	−0.52

BRI = Behavior Regulation Index, BRIEF-A = Behavior Rating Inventory of Executive Function—Adult version, CCI = Central Coherence Index, CWIT = Color Word Inference Test, MI = Metacognitive Index, GEC = General Executive Composite, RCFT = Rey Complex Figure Test, WCST = Wisconsin Card Sorting Test, * *p* = 0.05, ** *p* = 0.001 ^a^ Higher scores indicating more difficulties, ^b^ Higher score indicating better performance.

**Table 6:** Model-predicted means showing differences in change over time for primary outcomes from pre-treatment, post-treatment, and 6-month follow-up in the total ITT sample (*n* = 60).

			Model-Predicted Means				
Measure		*n*	Pre-Treatment (se)	Post-Treatment (12 Weeks) Follow-Up (se)	6-Month Follow-Up (se)	Difference in Change Over Time, Time Effect × Group Z-Value (*p*-Value) [Coefficient]	Time Effect 95% CI
EDE-Q							
Global							
	TCRT	30	4.17 (0.23)	3.98 (0.25)	3.90 (0.25)	0.51 (0.610) [0.13]	[−0.37, 0.63]
	Control	30	3.92 (0.23)	3.90 (0.24)	3.78 (0.24)		
	Group difference		−0.25 (0.33)	−0.08 (0.34)	−0.12 (0.35)		
BRIEF-A							
GEC							
	TCRT	30	138.43 (4.62)	131.43 (5.00)	126.07 (5.13)	2.22 (0.027) [14.13] *	[1.65, 26.60]
	Control	30	130.10 (4.62)	135.65 (5.00)	131.87 (5.06)		
	Group difference		−8.33 (6.54)	4.22 (7.07)	5.79 (7.21)		
BRI							
	TCRT	30	59.17 (1.92)	56.99 (2.04)	53.99 (2.09)	2.25 (0.024) [5.20] *	[0.68, 9.72]
	Control	30	57.43 (1.92)	58.55 (2.04)	57.46 (2.06)		
	Group difference		−1.73 (2.72)	1.56 (2.89)	3.47 (2.94)		
MI							
	TCRT	30	78.93 (2.70)	74.88 (2.85)	72.51 (2.90)	1.58 (0.113) [4.67]	[−1.11, 10.45]
	Control	30	76.00 (2.70)	76.96 (2.85)	74.24 (2.87)		
	Group difference		−2.93 (3.82)	2.08 (4.03)	1.74(4.07)		
EDI-3							
Drive for thinness							
	TCRT	30	21.74 (1.20)	21.42 (1.25)	19.38 (1.27)	1.63 (0.102) [2.22]	[−0.44, 4.88]
	Control	30	19.88 (1.19)	20.47 (1.25)	19.74 (1.29)		
	Group difference		−1.86 (1.69)	−0.96 (1.77)	0.36 (1.81)		
Bulimia							
	TCRT	30	8.51 (1.37)	7.94 (1.42)	6.58 (1.44)	0.29 (0.774) [0.40]	[−2.32, 3.12]
	Control	30	7.36 (1.36)	6.83 (1.42)	5.84 (1.45)		
	Group difference		−1.15 (1.93)	−1.10 (2.00)	−0.75 (2.04)		
Body dissatisfaction							
	TCRT	30	32.02 (1.59)	32.06 (1.65)	29.53 (1.67)	1.10 (0.273) [1.81]	[−1.42, 5.05]
	Control	30	30.89 (1.58)	32.17 (1.65)	30.21 (1.69)		
	Group difference		−1.13 (2.24)	0.11 (2.33)	0.68 (2.38)		
EDFLIX							
Total score							
	TCRT	30	94.00 (5.03)	100.49 (5.45)	107.76 (5.81)	−0.68 (0.497) [−5.44]	[−21.14, 10.25]
	Control	30	94.70 (5.03)	96.27 (5.53)	103.02 (5.71)		
	Group difference		0.70 (7.11)	−4.22 (7.76)	−4.74 (8.15)		
RCFT							
CCI							
	TCRT	30	1.22 (0.06)	1.26 (0.07)	1.46 (0.07)	0.34 (0.737) [0.04]	[−0.18, 0.26]
	Control	30	1.19 (0.06)	1.22 (0.07)	1.47 (0.07)		
	Group difference		−0.03 (0.09)	−0.04 (0.09)	0.01 (0.10)		
WCST							
Perseverative responses							
	TCRT	30	14.40 (1.73)	6.44 (1.96)	7.18 (2.05)	0.73 (0.467) [2.48]	[−4.21, 9.17]
	Control	30	11.00 (1.73)	9.12 (1.88)	6.27 (1.96)		
	Group difference		−3.40 (2.44)	2.68 (2.72)	−0.92 (2.83)		
Perseverative errors							
	TCRT	30	12.77 (1.39)	6.30 (1.57)	6.97 (1.64)	0.56 (0.57) [1.51]	[−3.75, 6.77]
	Control	30	10.13 (1.39)	8.37 (1.51)	5.84 (1.57)		
	Group difference		−2.63 (1.96)	2.07 (2.18)	−1.12 (2.27)		
CWIT							
Condition 3							
	TCRT	30	59.10 (4.02)	51.56 (4.18)	50.92 (4.24)	0.63 (0.528) [2.32]	[−4.90, 9.54]
	Control	30	50.33 (4.02)	45.43 (4.13)	44.48 (4.18)		
	Group difference		−8.77 (5.68)	−6.13 (5.88)	−6.44 (5.95)		

BRI = Behavior Regulation Index, BRIEF-A = Behavior Rating Inventory of Executive Function—Adult version, CCI = Central Coherence Index, CWIT = Color Word Inference Test, EDE-Q = Eating Disorder Examination Questionnaire, EDI-3 = Eating Disorder Inventory-3, EDFLIX = Eating Disorder Flexibility Scale, RCFT = Rey Complex Figure Test, TCRT = Transdiagnostic Cognitive Remediation Therapy, WCST = Wisconsin Card Sorting Test, * *p* = 0.05.

## Data Availability

Data are not openly available due to ethical/privacy restrictions as permission for data sharing was not provided by the participants or the Ethical Committee.

## Abbreviations

The following abbreviations are used in this manuscript:

AN	Anorexia nervosa
BAI	Beck anxiety inventory
BDI	Beck depression inventory
BED	Binge eating disorder
BN	Bulimia nervosa
BRI	Behavioral regulation index
BRIEF-A	Behavior rating inventory of executive function—adult version
CCI	Central coherence index
CRT	Cognitive remediation therapy
CWIT	Color word inference test
DOI	Duration of illness
DOT	Duration of treatment
D-KEFS	Delis–Kaplan executive function system
DSM-V	Diagnostic and statistical manual of mental disorders, 5th edition
ED	Eating disorder
EDE-Q	Eating disorder examination questionnaire
EDFLIX	Eating disorder flexibility index
EDI-3	Eating disorder inventory-3
GAI	General ability index
GEC	Global executive composite
IIT	Intention to treat
MI	Metacognition index
OSFED	Other specified feeding and eating disorder
RCFT	Rey complex figure test
RCT	Randomized controlled trial
TAU	Treatment as usual
TCRT	Transdiagnostic cognitive remediation therapy
WAIS-IV	Wechsler adult intelligence scale 4th edition
WCST	Wisconsin card sorting test
